# Nonhepatic Alagille Syndrome Associated With Predominant Cardioskeletal Anomalies: A Rare Case

**DOI:** 10.7759/cureus.17429

**Published:** 2021-08-25

**Authors:** Vishal V Bhende, Hardil P Majmudar, Tanishq S Sharma, Sohilkhan R Pathan, Deepakkumar V Mehta

**Affiliations:** 1 Pediatric Cardiac Surgery, Bhanubhai and Madhuben Patel Cardiac Centre, Shree Krishna Hospital, Anand, IND; 2 Pediatrics, Bhanubhai and Madhuben Patel Cardiac Centre, Shree Krishna Hospital, Anand, IND; 3 Medicine, Shree Krishna Hospital, Anand, IND; 4 Clinical Research, Bhanubhai and Madhuben Patel Cardiac Centre, Shree Krishna Hospital, Anand, IND; 5 Radiodiagnosis & Imaging, Pramukhswami Medical College, Karamsad, IND; 6 Radiodiagnosis & Imaging, Shree Krishna Hospital, Anand, IND; 7 Radiodiagnosis & Imaging, Bhaikaka University, Karamsad, IND

**Keywords:** predominant cardioskeletal anomalies, alagille syndrome, pediatrics, autosomal dominant genetic disorder, facial abnormalities

## Abstract

Alagille syndrome (ALGS) is a rare autosomal dominant genetic disorder with multisystem involvement including the liver, heart, skeleton, eyes, kidneys, and other organ systems, along with characteristic facial abnormalities. Some patients with ALGS may have isolated involvement of a particular system, such as a heart defect like the tetralogy of Fallot, an atrial septal defect (ASD), a characteristic facial appearance, or an isolated vertebral body anomaly. These individuals may or may not have liver anomalies or other features typically seen in the disorder. We report the case of a four-year-old female child with moderate ostium secundum ASD and branch pulmonary artery stenosis diagnosed since three months of age who presented with classical features of facial dysmorphism, posterior embryotoxon in the right eye, butterfly presentation of the T5 vertebra, delayed mental development, and history of recurrent infections. Bilateral branch pulmonary artery plasty with glutaraldehyde-treated pericardial patch and direct closure of the ASD leaving a patent foramen ovale was performed to correct the cardiac malformations. The surgery was uneventful without any postoperative complications. Currently, as no curative management of the disorder is available, the syndromic medical and surgical approach remains the mainstay in managing this condition.

## Introduction

First described in 1969 by Daniel Alagille, Alagille syndrome (ALGS) is an extremely rare autosomal dominant, multisystem disorder with a variable phenotypic presentation. The initial diagnostic criteria include the presence of intrahepatic duct scarcity and a minimum of the following three clinical features: chronic cholestasis, cardiac disease, skeletal abnormality, ocular abnormality, and specific facial characteristics. *JAGGED1* (*JAG1*) encodes the ligand Jagged1 in the Notch signaling pathway and is the predominant mutation responsible for ALGS. Approximately 90% of patients present with *JAG1* mutations and a minority of patients present with mutations in *NOTCH2*. Among the common features of ALGS, one is the association of congenital cardiac disease, with up to 94% of patients presenting with structural abnormalities, out of which 76% present with stenosis/hypoplasia of the branch pulmonary arteries, followed by the tetralogy of Fallot. Mortality increases significantly with cardiac involvement [1].

Here, we report an extremely rare case of ALGS in a four-year-old female child with associated ostium secundum atrial septal defect (ASD) and branch pulmonary artery stenosis.

## Case presentation

**Figure 1:**
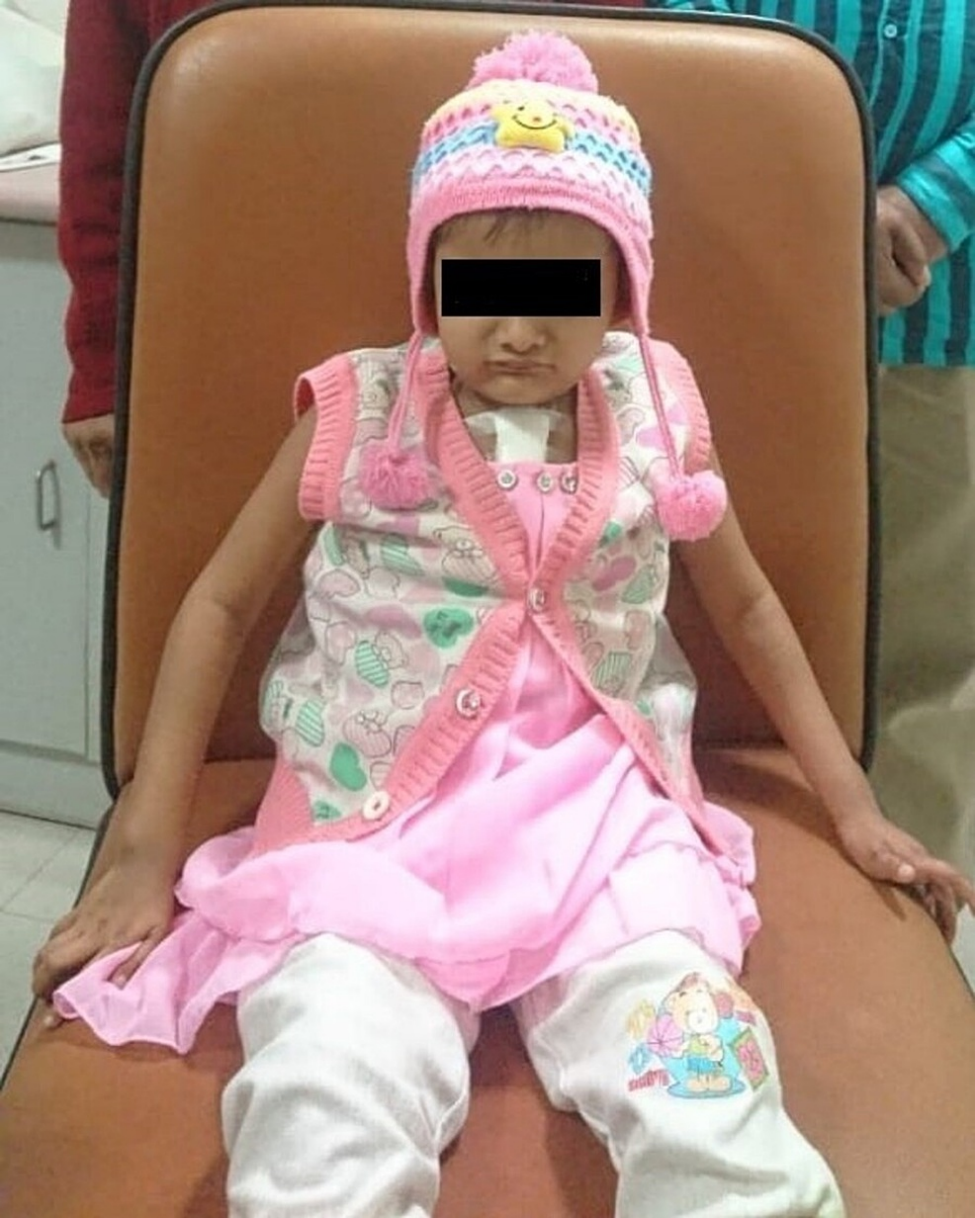
Facial dysmorphism features visible on the child.

**Figure 2:**
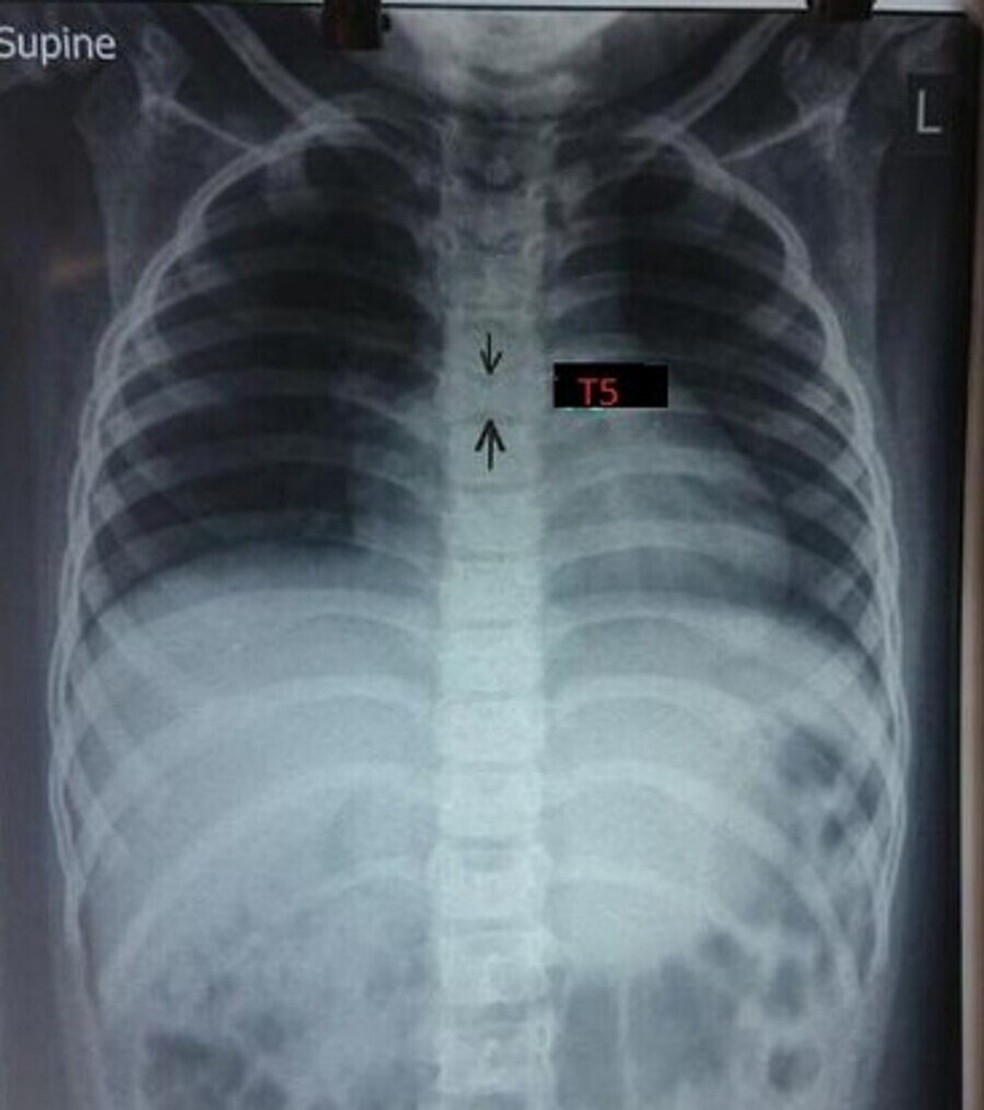
Chest X-ray showing butterfly presentation of the T5 vertebra.

**Figure 3:**
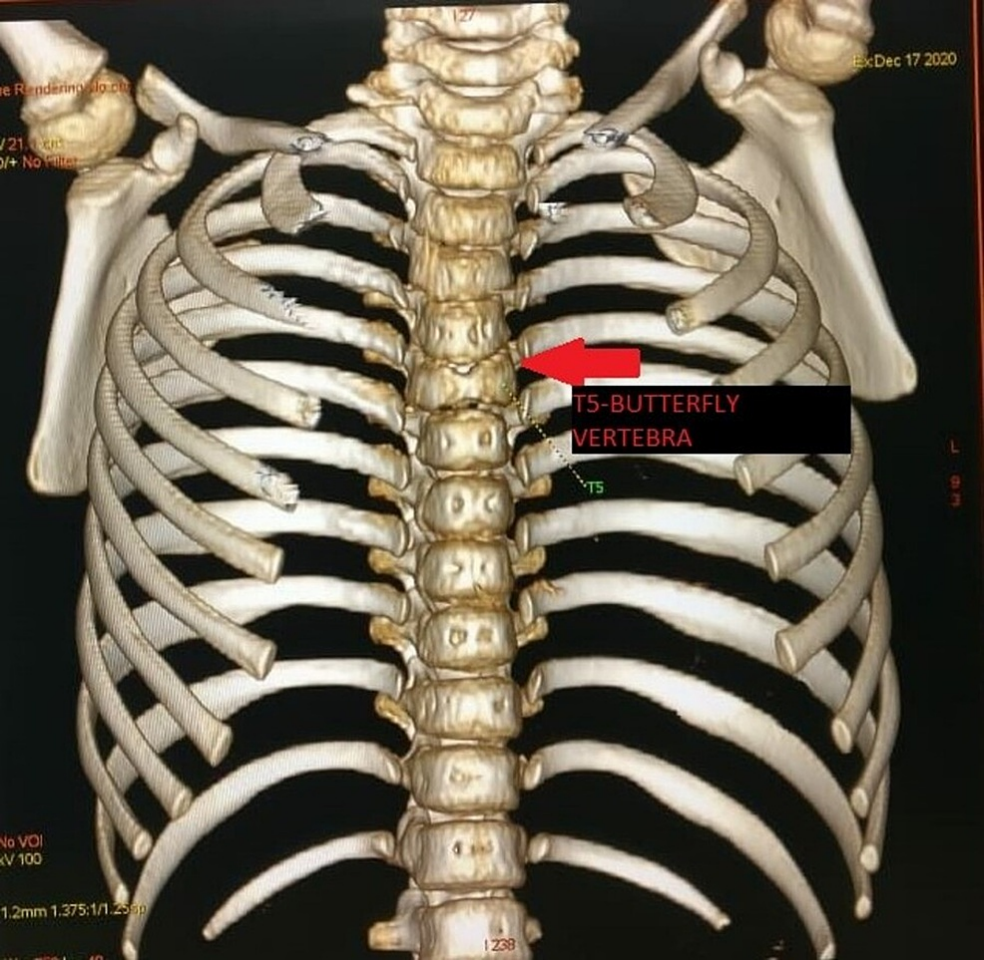
Three-dimensional reconstruction of the patient’s thoracic cage showing the T5 butterfly presentation.

A four-year-old female child with acyanotic congenital heart disease, that is, moderate ostium secundum ASD and branch pulmonary artery stenosis, since three months of age presented with classical features of facial dysmorphism such as frontal bossing, triangular facies, deep-set eyes, a pointed chin, and mild joint laxity (Figure 1). Delayed development was noted with mental development more delayed than motor development along with poor speech. Ocular examination revealed posterior embryotoxon and mild pigmentary retinopathy in the right eye. Chest X-ray showed abnormal segmentation in the T5 vertebrae (butterfly presentation) (Figures 2, 3) and past history was positive for recurrent infections and negative for neonatal cholestasis and maternal abnormalities. The above findings were highly suggestive of ALGS.

**Figure 4:**
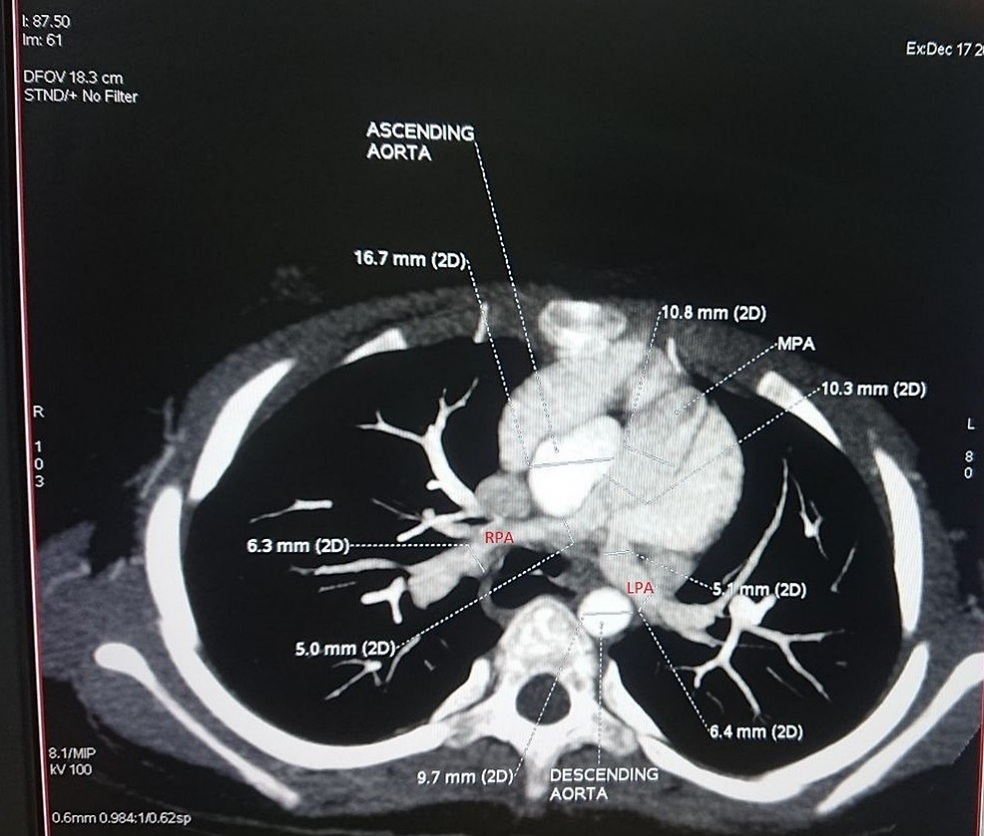
Right and left pulmonary artery stenosis depicted in the cardiac CT scan. CT: computed tomography

A confirmatory cardiac CT scan was performed which revealed a moderate ASD of ostium secundum type along with hypoplastic main right and left pulmonary arteries, with a McGoon ratio of 1.65 and Nakata index of 169.36 mm^2^/m^2^ (Figure 4). The common hepatic artery was seen arising from the proximal portion of the superior mesenteric artery, and a small vertical midline cleft in the body of the T5 vertebra, mainly in the posterior portion, was noted; these findings appeared to be congenital variants.

Further detailed evaluation and a preoperative color Doppler revealed severe bilateral branch pulmonary artery stenosis with moderate ostium secundum ASD and bidirectional shunting. Right ventricular pressure was significantly elevated with hypertrophy and dilatation of the right atrium and the right ventricle with normal biventricular function. Ultrasonography of the abdomen and pelvis reported no significant findings.

The patient was planned for bilateral branch pulmonary artery plasty with glutaraldehyde treated pericardial patch and direct closure of the atrial septal defect leaving a patent foramen ovale (PFO). The patient tested negative for severe acute respiratory syndrome coronavirus 2. She was operated on uneventfully and shifted to the Cardiac Surgical Intensive Care Unit on mechanical ventilation along with adrenaline and milrinone support. The patient was extubated on the third day after the surgery after 51 hours of Intensive Care Unit turnaround time. A postoperative echocardiogram color Doppler was performed one week after the surgery which revealed a severe obstruction in the left pulmonary artery flow with a peak gradient of 100 mmHg with diastolic tailing in the left pulmonary artery flow. In addition, it showed good flow in the proximal right pulmonary artery as well. Moreover, a small ostium secundum ASD with shunting from the right to left and severe right ventricle dysfunction with tricuspid annular plane systolic excursion of 5 mm were reported. Gradually, she was initiated on a liquid diet and started accepting full feeds. She was discharged in a stable condition with a healthy wound. She was also advised to follow proper wound care and hygiene and was scheduled for a follow-up.

## Discussion

ALGS, also known as arteriohepatic dysplasia/cardiovertebral syndrome, is an autosomal dominant disorder presenting with multisystem abnormalities. It can affect the liver, heart, skeleton, eyes, kidneys, and central nervous system and can present with characteristic facial features. First reported by Daniel Alagille in 1969, ALGS has a prevalence of 1:70,000 based on the presence of neonatal liver disease. However, this prevalence is highly underestimated due to the lack of genetic testing and family studies. *JAG1* mutations account for 97% of the cases whereas approximately 1% of the cases are due to *NOTCH2* mutations [2]. Our patient presented with ostium secundum ASD and severe bilateral branch pulmonary artery stenosis, with a negative history of neonatal cholestasis or hepatic complications. This was consistent with the study reported by Turnpenny and Ellard [2].

Cardiac symptoms are accompanied by the presence of characteristic facial features such as frontal bossing, triangular facies, deep-set eyes, a pointed chin, and mild joint laxity. Mitchell et al. reported typical features such as triangular presentation, a broad forehead, deep-set eyes, moderate hypertelorism, a pointed chin, and the bulbous tip of the nose, all of which were seen in our case. However, these features are not confirmatory and tend to vary with considerable observer bias [1].

Nischal et al. reported ocular findings among ALGS patients. They concluded that, in ALGS patients, optic disc drusen in one eye was evident in 95% of the patients, and 80% of patients presented with bilateral disc drusen. The most prominent ocular finding among ALGS patients was posterior embryotoxon and a prominent anterior Schwalbe’s line, which was visible in about 88% of ALGS patients compared to 8-15% of the normal population [3]. In our case, on ophthalmic evaluation, posterior embryotoxon and mild pigmentary retinopathy were evident in the right eye. These findings were consistent with the study by Nischal et al. and highlight the importance of multisystem evaluation in ALGS patients.

Skeletal abnormalities in the form of characteristic vertebral segmentation anomaly, also known as butterfly vertebrae, are a classic presentation among ALGS patients. The presence of a sagittal cleft in one or more thoracic vertebrae due to the failure of anterior vertebral arch fusion is usually visible on an anteroposterior radiograph [1]. In our patient, on anteroposterior chest radiographs, a classic segmental cleft (butterfly vertebrae) was evident in the T5 vertebrae. Mild joint laxity was also prominent. These features were consistent with the classic criteria for ALGS.

Vascular abnormalities are a fairly common presentation among ALGS patients, and pulmonary artery involvement is the most common manifestation. In a study by Kamath et al., 14% of the patients presented with intracranial bleeding and accounted for 25% of the mortality. Our patient had no history of intracranial bleeds but presented with severe bilateral pulmonary artery stenosis. This vascular involvement was attributed to the notch signaling pathway disturbance, and the involvement of *JAG1* was predominant in disrupting the vasculogenesis process [4].

Because systemic evaluation revealed no renal involvement, ALGS type 1 was suspected in our case, supporting the finding that the positive impact of *JAG1 *mutation leads to the predominance of cardiac symptoms [1,2,5]. The frequency of renal involvement has been attributed to the presence of *NOTCH2 *mutations or ALGS type 2 [5].

The presence of a higher frequency of motor delay among these patients is thought-provoking. Approximately 16% of the cases are affected by the motor delay, attributed to large genetic mutations [6]. Tilib Shamoun et al., in their study on immune dysregulation among ALGS patients, identified the presence of impaired T-cell response and explained the predisposition of such patients to conditions such as asthma, eczema, food allergies, airway atopy, and otitis media. Hence, an impaired T-cell response may be responsible for the recurrent infections in our patient [7].

Our patient was planned for bilateral branch pulmonary artery plasty with glutaraldehyde-treated pericardial patch and direct closure of the ASD leaving a PFO to counter the cardiac anomalies. The postoperative period was uneventful and the patient showed symptomatic improvement. Supportive treatment is the only therapeutic modality at present. Modulation of the notch signaling pathway, cell-based therapies, or correction of specific mutations in vitro or in vivo are some of the newer therapies to explore in the future [1].

## Conclusions

Higher clinical suspicion is crucial to diagnose such rare disorders. The focus should be on the classic criteria and to evaluate the patient based on the protocol to confirm the presence of ALGS based on clinical characteristics, as well as to rely less on genetic testing. Multisystem management, monitoring, and family assessment must be conducted to not miss out on the diagnosis of ALGS. ALGS may present with hepatic complications or cardiac anomalies in isolation; therefore, it is important to not miss out on the diagnosis and to have a higher clinical suspicion for ALGS. Although current therapeutic approaches are supportive, in the future, interventions to modulate the gene signaling pathway and to prevent the mutation via the identification of a specific window during the embryogenesis phase need to be developed. In addition, genetic engineering and targeted pharmacologic agents can help augment the notch signaling pathway to prevent such deformities and reduce the incidence.
